# Co-expression of the *RPS6KB1* and *PDPK1* genes for production of activated p70S6K1 using bac-to-bac baculovirus expression system

**DOI:** 10.1007/s11033-024-10136-0

**Published:** 2025-01-17

**Authors:** Anna Bdzhola, Oksana Malanchuk, Sergii Palchevskyi, Ivan Gout, Valeriy Filonenko, Alexander Zhyvoloup

**Affiliations:** 1 Department of Cell Signaling, Institute of Molecular Biology and Genetics, National Academy of Sciences of Ukraine, Kyiv, 03143 Ukraine; 2 Department of Structural and Molecular Biology, University College London, London, WC1E 6BT UK

**Keywords:** Protein expression, Protein phosphorylation, S6K1, PDPK1, Baculovirus expression system, Kinase activity

## Abstract

**Background:**

Ribosomal protein S6 kinase 1 (p70S6K1) is a member of the AGC family of serine/threonine kinases which plays a role in various cellular processes, including protein synthesis, cell growth, and survival. Dysregulation of p70S6K1, characterized by its overexpression and/or hyperactivation, has been implicated in numerous human pathologies, particularly in several types of cancer. Therefore, generating active, recombinant p70S6K1 is critical for investigating its role in cancer biology and for developing novel diagnostic or therapeutic approaches.

**Methods:**

The baculovirus dual expression system was utilized, enabling the co-expression of two recombinant proteins in infected cells: (a) His-tagged S6K1 with a deletion of the C-terminal autoinhibitory motif and a phosphomimetic mutation at the mTORC1 phosphorylation site (T389D), and (b) untagged PDPK1 lacking the PH domain. The high activity of the purified kinase was confirmed by immunoblotting, as well as by Kinase-Glo and AlphaScreen kinase assays.

**Results:**

Efficient expression of both recombinant proteins was achieved, resulting in highly pure preparations of His-tagged p70S6K1. The high activity of the purified kinase was confirmed through multiple kinase assays, demonstrating significantly higher levels of substrate phosphorylation compared to the tested commercial product.

**Conclusion:**

Here, we report a reliable and efficient methodology for the expression and purification of highly active p70S6K1 (His-actS6K1) in quantity and quality that is suitable for biochemical/biophysical studies and high-throughput enzymatic assays. Our developed methodology offers a rapid and cost-effective approach for producing constitutively active His-actS6K1, which can be utilized in academic research and biotechnology.

**Supplementary Information:**

The online version contains supplementary material available at 10.1007/s11033-024-10136-0.

## Introduction

S6 Kinase 1 (S6K1), encoded by the *RPS6KB1* gene, is a key member of the AGC family of serine/threonine kinases, known for its implication in cell growth, proliferation and survival [1]. Among three isoforms expressed via the use of alternative translational start codons (p85, p70, p60 [2, 3]), p70S6K1 has been identified as dominant and the only isoform regulated by growth factors. Due to its key roles in protein synthesis and cell cycle regulation, p70S6K1 has been implicated in a vast number of human diseases, such as obesity, diabetes, and cancer. Upregulated p70S6K1 activity was observed in patients with cervical [4], colorectal [5], liver [6], gastric [7], and gallbladder [8] cancers, among other types. There are also various facets of cancer progression that could be influenced by upregulation of p70S6K activity or its constitutive activation, such as cancer stemness, the epithelial-mesenchymal transition [9–11], and drug resistance [12]. Consequently, the selective inhibition of constitutively active p70S6K1 is currently being investigated as a promising approach for treating associated metabolicdiseases [13–15].

The process of S6K activation is a complex mechanism involving multi-site phosphorylation [16]. This mechanism involves two critical steps: phosphorylation of the hydrophobic motif (HM) at T389 by mTORC1 and phosphorylation of the T229 site in the kinase T-loop by PDPK1. It has been found that in its inactive conformation, the C-terminal autoinhibitory domain (AID) of S6K1 sterically blocks access to the HM and T-loop, thereby suppressing kinase activation. In response to elevated levels of growth factors, cytokines, and mitogens, multiple phosphorylation events occur within the AID region, leading to conformational changes that expose critical T389 and T229 residues in the HM and T-loop, respectively, priming them for phosphorylation by upstream regulators [17]. Therefore, these three consecutive steps are required for S6K1 to enter the active mode and to proceed with its functions as a kinase. Emerging evidence has shown that HM phosphorylation is not necessary for PDPK1 to phosphorylate S6K1 at the activation loop; instead, activation loop phosphorylation is necessary for mTOR to phosphorylate the HM. Once phosphorylated by PDPK1, S6K1 is stabilized in its active conformation within cells [18, 19].

Previously the expression and purification of the T-loop phosphorylated and constitutively active p70S6K1 form have been reported in HEK293 cells via transient transfection [20] and in Sf9 cells through co-infection with two recombinant viruses encoding S6K1 and PDPK1 constructs [21, 22]. However, Western blot analysis demonstrated minimal T229 phosphorylation and catalytic activity in the baculovirus-derived kinase.

In this article, we present an evolved baculovirus-based method for producing a highly active form of p70S6K1. We exploited a novel approach to achieve efficient in situ phosphorylation of recombinant S6K1 at T229. Using the Bac-to-Bac™ Baculovirus Expression System, we generated a dual baculovirus vector encoding both *RPS6KB1* and *PDPK1*. This construct enabled the synthesis of both proteins in each infected cell at a stable ratio, resulting in efficient T229 phosphorylation and high expression of active p70S6K1. Additionally, we developed a simple, robust, and efficient protocol for the expression, purification, and testing of highly active S6K1in sufficient quantity and quality, suitable for high-throughput enzymatic assays.

## Materials and methods

### Reagents and chemicals

Commercial p70S6K1 was purchased from Enzo Life Sciences (#SE-345, active, 69 U/mg, 0.1 mg/ml). Phospho-p70S6K1 (T229) antibody (Fisher Scientific, #10727703), phospho-rpS6 (S235/236) antibody (Cell Signaling, #5364), phospho-rpS6 (S240/244) antibody (Cell Signaling, #2215), goat anti-Rabbit IgG Alexa Fluor™ Plus 800 conjugate (Fisher Scientific, #15667898), and goat anti-rabbit IgG HRP conjugate (Promega, #W4018) were used. The biotinylated substrate-peptide for p70S6K1, corresponding to residues 225–249 of the rpS6 protein (Biotin-QEQIAKRRRLSSLRASTSKSESSQK) was obtained from Mimotopes (Melbourne, Australia). Oligonucleotides (Table S1) were ordered from Eurofins Genomics (Ebersberg, Germany). AlphaScreenTM Rabbit IgG Detection Kit, Revvity, # 6,760,607 C. S6K inhibitors included LY2584702 (Merck, # SML2892) and Staurosporine (Thermo Fisher Sci, # 62996-74-1). Pierce™ ECL Western Blotting Substrate (Fisher Scientific, #32106), imidazole (Millipore, Watford, UK), glutathione resin (CLONTECH Laboratories, USA), and Ni-NTA agarose (Qiagen, Germany, # 30210) were also used. All other chemicals, salts, and buffers were purchased from Sigma, Inc. (St. Louis, MO).

### Construction of the recombinant vector and recombinant baculovirus

A truncated gene for human 3-phosphoinositide dependent protein kinase 1 (*PDPK1* gene) was PCR-amplified using the forward primer AZ001 and the reverse primer AZ002 (Table S1). The primers were designed to amplify isoform 2 of the kinase gene, lacking the membrane-binding PH (pleckstrin homology) domain (residues 51–359). The amplified DNA was cloned into NcoI and PvuII sites of the pFastBac-DUAL vector (Invitrogen). The resultant intermediate construct, pFastBac-DUAL: PDPK1(ΔPH), was then used for cloning cDNA for human S6K1 (*RPS6KB1* gene). The DNA encoding S6K1 (residues 3-398) was PCR-amplified using the forward primer AZ003 and the reverse primer AZ005 (Table S1). The primer AZ003 was designed to introduce a 6xHis-tag and TEV cleavage site (ENLYFQ|G) at the N-terminus of the gene product, while AZ005 was designed to truncate the autoinhibitory domain (AID) of the S6K1 protein. The template DNA contained the T389D mutation, mimicking mTORC1-dependent phosphorylation of S6K1. The amplified DNA was cloned into the BamHI and EcoRI sites of the intermediate pFastBac-DUAL: PDPK1(ΔPH) construct. The modified PDPK1 and S6K1 were encoded by theresultant construct, pFastBac-DUAL: PDPK1(ΔPH): S6K1(ΔAID, T389D) and their expression was controlled by P10 and Ppol promoters respectively. The dual construct was used to generate the recombinant baculovirus following the standard protocol for Bac-to-Bac™ (Thermo Fisher Scientific, #10359016) expression system.

### Insect cell culture

Sf9 (*Spodoptera frugiperda*) insect cells were cultured either as adherent cells in T25 flasks or as suspension cultures in conical baffled flasks with constant shaking at ~ 120 rpm at + 27 °C. The Insect-XPRESS™ medium (Lonza, # BELN12-730Q) was supplemented with 10% fetal bovine serum (FBS) and 1x Pen/Strep antibiotics (Cytiva, #SV30010).

### Insect cell infections and His-actS6K1 protein expression

Virus generation was conducted following the Bac-to-Bac™ Baculovirus Expression System user guide. Briefly, bacmid DNA was isolated from Lac-negative DH10Bac clones transfected with the dual construct encoding PDPK1-ΔPH and S6K1(ΔAID, T389D), later termed as “His-actS6K1”. Sf9 cells and the transfection reagent Escort™ IV (Sigma, #L3287) were used to generate the primary recombinant baculovirus. A total of 2.5 × 10^6^ cells were seeded onto T25 flasks and allowed to attach at + 27 °C for at least 1 h. Escort™ IV and bacmid DNA were diluted separately in blank medium and combined to form lipid-DNA cell-permeating complexes, which were incubated at RT for 30 min. The transfection mixture was then overlaid onto the cells and incubated at + 27 °C. After 5–7 h of incubation, the transfection medium was replaced with fresh Insect-XPRESS™ medium containing antibiotics. After 72 h of incubation, both non-infected control cells and infected Sf9 cells were harvested and tested for His-actS6K1 expression. The baculovirus containing medium was collected and stored at + 4 °C for further infections. For routine production of His-actS6K1, Sf9 cells at a density of 2 × 10^6^ cells/ml were infected at a multiplicity of infection (MOI) of 4–5 and incubated at + 27 °C in a conical baffled flask at 120 rpm. The cells were harvested 48 h post-infection by centrifugation at 1500 rpm for 20 min at + 4 °C. The baculovirus titers were determined using a traditional virus plaque assay (agarose overlay with neutral red staining) and the FastPlax Titer Kit (Novagen, # 70850), with findings indicating that protein yield (His-actS6K1) from a bicistronic S6K1/PDPK1 virus was influenced primarily by post-infection incubation duration rather than multiplicity of infection (MOI).

### Purification of recombinant His-actS6K1 protein using Ni-NTA affinity chromatography

Cell pellets from non-infected (control) and baculovirus-infected Sf9 cells were resuspended in ice-cold Lysis Buffer containing 20 mM Tris–HCl (pH 8.0), 200 mM NaCl, 2 mM MgCl_2_, 2 mM NaF, 10% glycerol, 10 mM imidazole, 1% Nonidet P40, 1 mM PMSF, 50 mM β-glycerophosphate, 1x cOmplete Protease Inhibitor Cocktail (EDTA free, Roche, #11873580001), and 25 U/ml benzonaze. All subsequent purification steps were carried out at + 4 °C, unless otherwise specified. Cells were resuspended in ~ 25 pellet volumes (PV) of Lysis Buffer and incubated on ice for 30 min with periodic vortexing. The soluble fraction was separated from the pellet by centrifugation for 30 min at 48 000 g. The supernatant was collected and loaded onto Ni-NTA agarose, with 0.25 PV of the resin pre-equilibrated with Lysis Buffer in a gravity-flow column before use. The supernatant-loaded column was washed with 50 PV of Washing Buffer (20 mM Tris–HCl (pH 8.0), 200 mM NaCl, and 20 mM imidazole). The bound proteins were eluted using ~ 1.5 PV of Elution buffer (Washing Buffer supplemented with 350 mM imidazole). The purified recombinant protein was dialysed twice using Spectra/Por^®^ 3 dialysis membrane with a molecular weight cut off (MWCO) at 3.5 kDa (Thermo Scientific) against at least 100 volumes of 50% glycerol, 20 mM Tris–HCl (pH 7.5), 200 mM NaCl, 10 mM DTT, 0.1 mM EGTA, and 0.03% Brij-35.

### Expression and purification of recombinant GST-rpS6Ct protein using GST-beads affinity chromatography

Electrocompetent *Escherichia coli* BLR(DE3) cells were transformed with the pET42a vector containing ribosomal protein S6 C-terminal sequence (residues 208–249) conjugated with a GST-6xHis-tag, referred to as GST-rpS6Ct. The cells were grown at + 37 °C in Luria Broth medium until the OD_600_ reached 0.6–0.8. The cells were then induced with 0.5 mM IPTG and incubated for 3 h at + 25 °C. The cell suspension was centrifuged 20 min at 6200 g in a pre-cooled (+ 4 °C) Beckman JLA 8.1000 rotor, and the pellet was resuspended in Lysis Buffer containing 20 mM Tris HCl (pH 8.0), 250 mM NaCl, 1mM β-mercaptoethanol, 3 mM MgCl_2_, 5 mM imidazole, 50 µg/mL deoxyribonuclease I (DNaseI Bovine pancreas), and 1x PIC (cOmpleteTM Mini Protease Inhibitor Cocktail— Roche, EDTA free). The cell suspension was then sonicated using Soniprep150 (15 cycles) and pelleted by centrifugation for 30 min at 14 000 rpm. Supernatants containing GST-rpS6Ct were collected and loaded onto pre-equilibrated glutathione resin in Washing Buffer (20 mM Tris HCl (pH 8.0), 250 mM NaCl, 1mM MgCl_2_) and incubated in-batch for 1 h at + 4 °C. The column was then washed 5 times with 50 PV of Washing Buffer. Bound proteins were eluted using Elution Buffer containing 50 mM Tris-HCl (pH 8.0), 10% glycerol, and 10 mM reduced Glutathione. Two-step dialysis of purified recombinant protein was conducted against 50 mM Tris-HCl (pH 8.0), 150 mM NaCl and 50% glycerol buffer using the Spectra/Por^®^ 3 dialysis membrane with a MWCO of 3.5 kDa.

### Immunoblotting assay using Anti-Phospho-rpS6 (Ser240/244) and Anti-Phospho-rpS6 (Ser235/236) antibodies

The specific protein kinase activity of recombinant His-actS6K1 was tested using recombinant GST-rpS6 (residues 208–249) as a substrate. The reaction conditions were: 200 µM ATP, 660 nM GST-rpS6(С-term), and 0.1 to 20 nM recombinant p70S6K1. The Assay Buffer consisted of 4 mM MOPS (pH 7.2), 2.5 mM β-glycerophosphate, 1 mM EGTA, 0.4 mM EDTA, 4 mM MgCl_2_, and 0.5 mM DTT. All analyzed enzyme samples were pre-diluted with the reaction buffer at a 1:10 ratio and 1 µl of the dilution was added per reaction. The commercial standard was applied without dilution. The total volume of the reaction was 50 µl. For testing the enzymatic activity of His-actS6K1 preparations in serial dilutions, the complete reaction mix was diluted 1:5 using the substrate/ATP premix in Assay Buffer. The reactions were conducted at + 37ºC for 15 min, and Laemmlie buffer was added to stop the reaction. The phosphorylation level of the protein substrate was assessed by PAGE and Western blotting using anti-Phospho-RPS6 (S240/244) antibodies. The phosphorylation level was then evaluated using the HRP-conjugated secondary antibodies and an ECL kit. The immunospecific chemiluminescence signal was visualized using X-ray film and scanned by Odyssey XF for further quantification using the Image Studio program. Analysis of substrate phosphorylation using anti-Phospho-RPS6 (S235/236) antibodies was conducted under the same reaction conditions, except 100 nM of His-actS6K1 was used in the reactions, and the secondary antibodies were goat anti-Rabbit IgG Alexa Fluor™ Plus 800 Conjugate. The Western blot membrane was scanned using Odyssey CLx Imager.

### Kinase activity assay using Kinase-Glo kit (Promega, #V6711)

The specific protein kinase activity of recombinant His-actS6K1 was tested using a custom-ordered synthetic peptide Biotin-QEQIAKRRRLSSLRASTSKSESSQK, corresponding to residues 225–249 of the rpS6 protein. The reaction conditions were as follows: 1 µM ATP, 100 µM peptide, and 0.1 to 140 nM recombinant S6K1. The Assay Buffer consisted of 20 mM MOPS (pH 7.2), 2.5 mM β-glycerophosphate, 5 mM MgCl_2_, 0.05% BSA, 0.05% Brij-35, and 5 mM β-mercaptoethanol. The reaction was conducted at + 30^º^C for 30 min. The enzymatic activity was measured using the Promega Kinase-Glo assay kit (# V6711) according to the manufacturer’s protocol. Briefly, the assay plate was equilibrated at RT for ~ 5 min, then an equal volume of Kinase-Glo reagent was added to the reaction wells, and the plate was incubated for 2 min at RT before signal reading. ATP-dependent chemiluminescence was measured using a Clariostar plate reader.

### Kinase activity assay using AlphaScreen ™ (Perkin Elmer, #6760607)

An investigation was conducted to determine whether Staurosporine and the S6K1-specific inhibitor LY2584702 inhibited His-actS6K1 activity. The assay was developed in a 384-well plate format. The kinase reaction conditions were as follows: 25 mM MOPS (pH 7.2), 5 mM β-glycerophosphate, 5 mM MgCl2, 1 mM β-mercaptoethanol, 0.2 mM EGTA, 0.05% BSA, 0.01% Brij-35, 0.3 ng/µl enzyme, 50 nM peptide (biotin-QEQIAKRRRLSSLRASTSKSESSQK), and 10 µM ATP. The reaction was performed at RT for 2 h, and an equal volume of Signal Detection mix was added to the reaction for further RT incubation for 3 h. The signal Detection mix contained: 25 mM MOPS (pH 7.2), 12.5 mM EDTA, 0.05% BSA, 1% Tween-20, 10 ng/ml (each) Donor and Acceptor beads, and 1:1500 dilution of antibody (S240/244 Phospho-rpS6 Cell Signaling). The signal was measured using the EnVision plate reader.

### Protein gel electrophoresis and Western blot analysis

Samples from cell lysates and eluates were prepared using 5x Laemmlie buffer, then heated at + 95 °C for 5 min and loaded onto a gel. SDS-PAGE was performed under reducing conditions using 4–20% Bis-Tris Precast Gel (Millipore, Germany) in MOPS running buffer (Millipore, Germany) and stained with InstantBlue^®^ Coomassie (Abcam, USA) or transferred onto a LF-PVDF membrane (Millipore, Germany) using a semi-dry blotting approach in Trans-Blot Turbo transfer buffer (Bio-Rad Lab., UK). After blocking the membrane in buffer (Li-COR Biosciences, USA) for 30 min, it was probed with the Phospho-p70 S6 Kinase (T229) polyclonal antibody (Invitrogen, UK), Phospho-S6 Ribosomal Protein (pS235/236), or Phospho-S6 Ribosomal Protein (pS240/244) according to the manufacturer’s instructions. The signals were detected using the Odyssey CLx Imager (Li-COR Biosciences, USA) or X-ray film, depending on the nature of the secondary antibody used.

## Results

### Cloning and construction of recombinant baculovirus

**Fig. 1:**
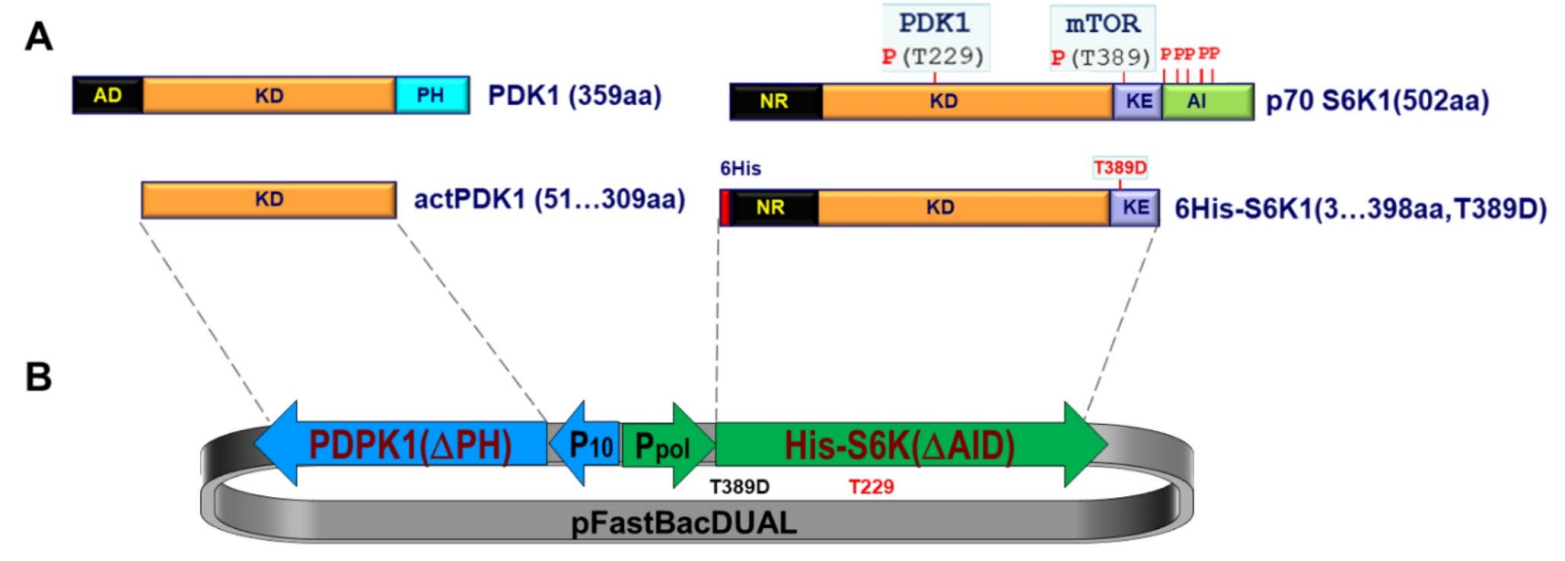
Schematic diagram of recombinant baculovirus construction. **A**) Domain structure of wild type and recombinant PDPK1 and p70S6K1; **B**) Construction scheme of the recombinant plasmid co-expressing modified p70S6K1 along with PDPK1 in the pFastBac™ Dual vector

To facilitate co-expression of the target proteins, a recombinant baculovirus system was constructed using a vector possessing two multiple cloning sites, controlled by the polyhedrin (PH) and p10 promoters, enabling the simultaneous expression of two heterologous genes. For the production of T-loop phosphorylated p70S6K1, we utilized an insect expression system, which offers high levels of protein expression with post-translational modifications similar to those in mammalian cells. Therefore, we first constructed the pFastBac™ Dual plasmid loaded with the 6His-p70S6K1(ΔAID)-T389D insert under the control of Ppol promoter together with the PDPK1(ΔPH) insert controlled by the p10 promoter (Fig. 1B). The schematic structure of the recombinant product is shown in Fig. 1A. In this construct, the native form of p70S6K1 was modified by adding a 6His-tag to the N-terminal regulatory domain (NR), deleting the C-terminal domain containing the autoinhibitory motif (AID), and mutating the T389 site to D389 to mimic phosphorylation by mTORC1. The resultant recombinant plasmid was used to clone 6xHis-tagged S6K1 (residues 3-398, T389D) under the control of the Ppol promoter, along with untagged PDPK1, lacking the membrane-binding pleckstrin homology (PH) domain (residues 51–359), under the control of the p10 promoter (Fig. 1B). The truncated variant of PDPK1, no longer restricted to the inner membrane surface, was expected to increase S6K1 activation efficiency.

PDPK1-dependent phosphorylation at T229 is a critical regulation point for S6K1 activation that cannot be phosphomimicked. While the phosphorylation-mimicking T389D mutation and removal of the autoinhibitory domain were necessary, these modifications alone were not sufficient to achieve high S6K1 activity. It has been observed that the S6K1 activity of the double mutant (T389E, T229E) protein, expressed from baculovirus, remained low [21]. Therefore, to achieve high S6K1 activity, T229 must be phosphorylated.

### Expression and purification of the His-actS6K1

**Fig. 2:**
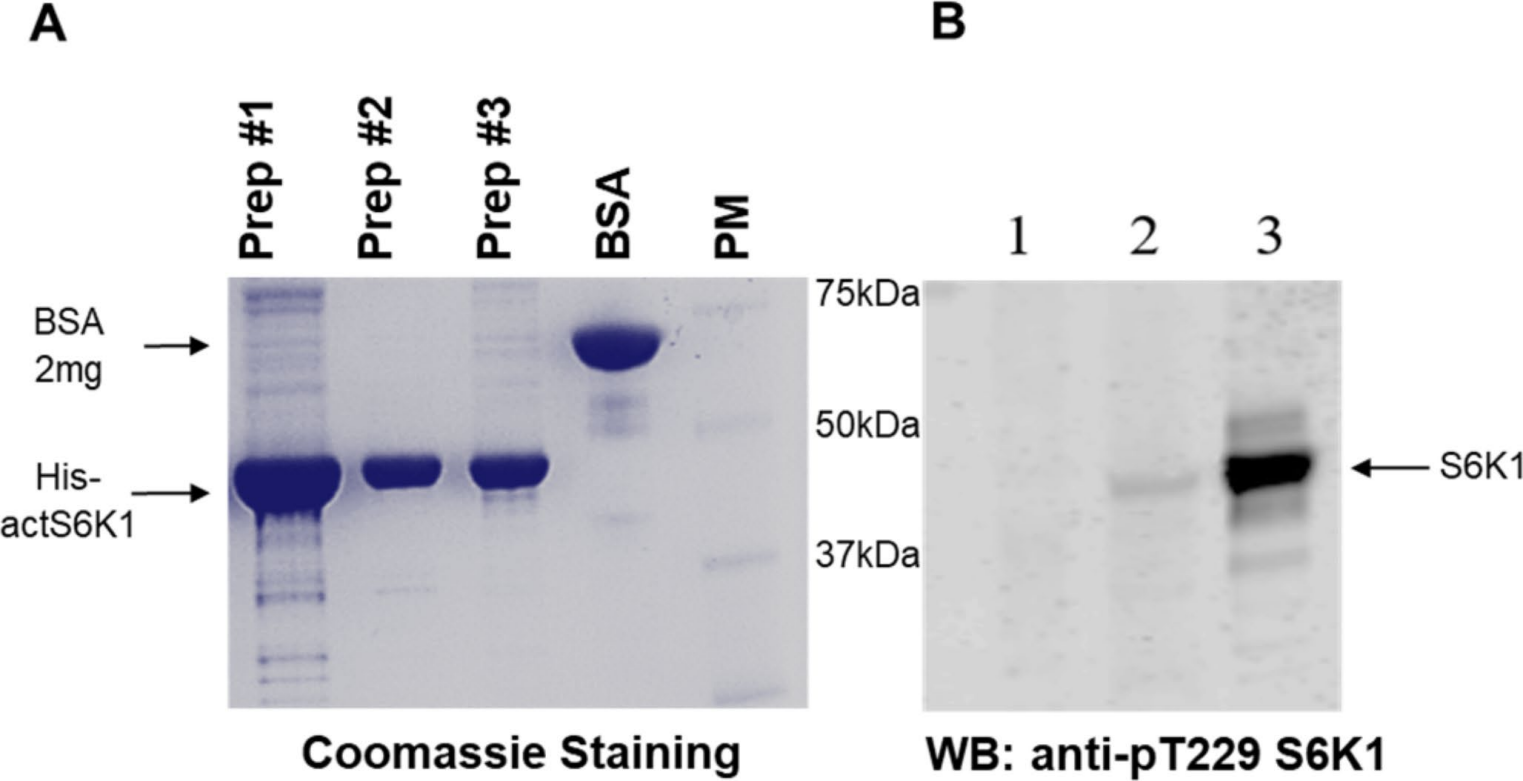
His-actS6K1 was efficiently expressed from the recombinant baculovirus and phosphorylated at the PDPK1-specific site T229. **A**) SDS-PAGE of purified His-actS6K1 protein stained with Coomassie InstantBlue. The samples from the final dialysis of three preparative expressions/purifications were separated on the gel; **B**) Western blot analysis of purified His-actS6K1 T229 phosphorylation using the phospho-T229 antibody. 1 – lysate (20 µg) of non-infected Sf9 cells; 2 – lysate (20 µg) of Sf9 cells infected with recombinant His-actS6K1 baculovirus; 3 – purified His-actS6K1 (1 µg). Positions and values of the protein marker are applicable for both **A**) and **B**)

Using the conventional Sf9 suspension cell culture method, single step Ni-NTA affinity chromatography, and subsequent dialysis, we obtained a highly pure recombinant product with a molecular weight of ~ 45 kDa (Fig. 2A, Prep 1, 2, 3), corresponding to the calculated molecular mass of His-actS6K1 (46,4 kDa). As represented in Fig. 2B, Western-blot analysis using specific anti-pT229 S6K1 antibodies confirmed the presence of the correctly sized protein in 20 µg of total lysate from infected Sf9 cells (lane 2), and 1 µg of purified recombinant His-act S6K1 (lane 3). The recombinant protein was not detected in the 20 µg of total lysate from non-infected cells (lane 1). The immunoblot shown in Fig. 2B, demonstrated not only the specificity of the obtained protein but also its effective phosphorylation at its T229 site by co-expressed PDPK1(ΔPH), which is essential for S6K1 activation. The protein yield, concentration of the final samples, and enzymatic activity are summarized in Table S2.

### Activity assay of the purified His-actS6K1

**Fig. 3:**
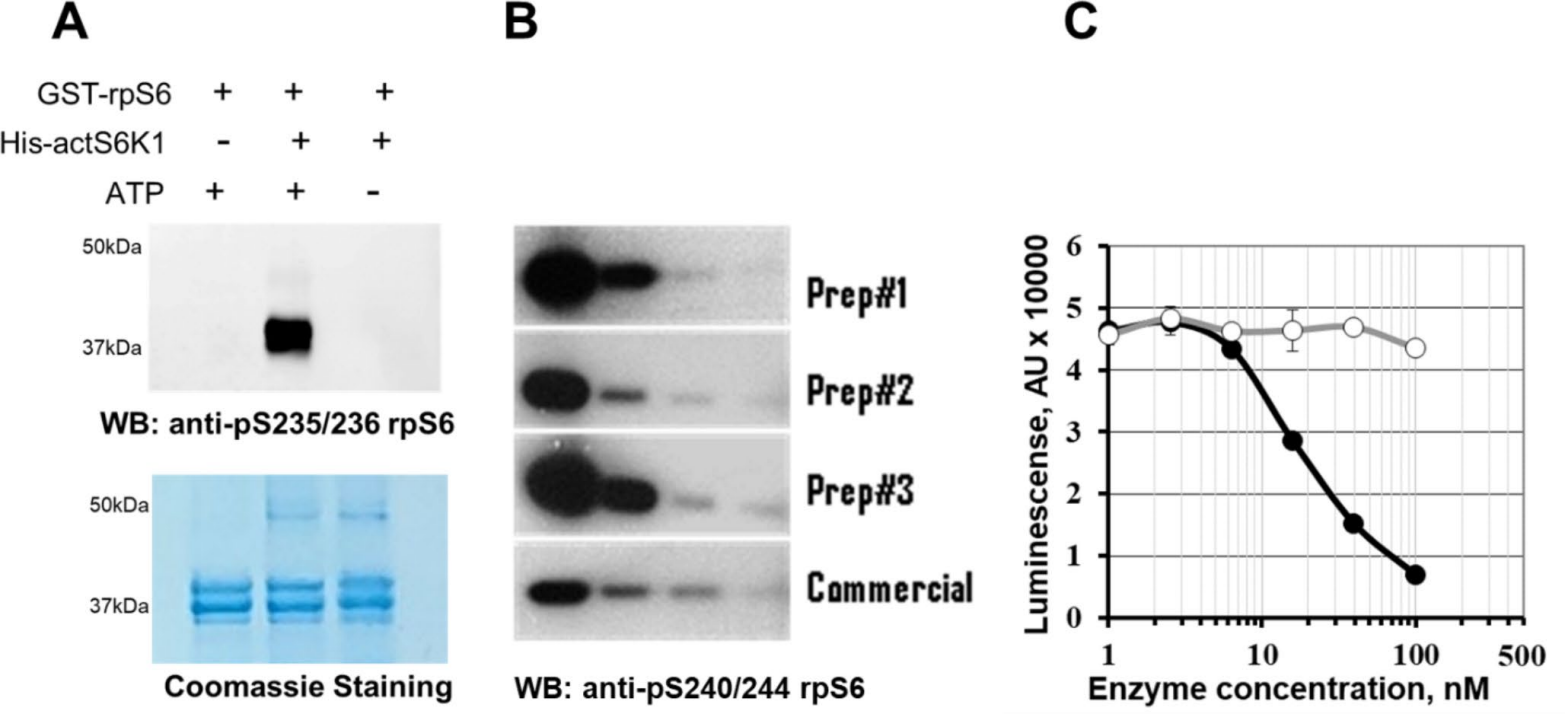
His-actS6K1 enzymatic activity was characterized using different substrates and assay platforms. **A**) Phosphorylation of recombinant GST-rpS6 protein tested with the phospho-rpS6 (S235/236) antibody; **B**) Kinase activity of His-S6K1 preparations and commercial S6K1 were tested in the serial 1:5 dilutions using the same substrate and phospho-rpS6 (S240/244) antibody **C**) Kinase activity in the presence (black circles) or absence (open circles) of the rpS6 substrate peptide (synthetic peptide Biotin-QEQIAKRRRLSSLRASTSKSESSQK) tested using the Kinase-Glo assay

To further investigate the activity of the expressed protein, several kinase assays were performed (Fig. 3). The results shown in Fig. 3 (A, B) demonstrate the levels of ribosomal protein S6 phosphorylation by the purified His-actS6K1 at various sites, as detected by different antibodies (pS235/236 and pS240/244). The preparations (preps) obtained from the co-expression approach displayed significantly higher activities as compared to the commercial enzyme (Fig. 3B). The average activity of the three preps was 137.9 ± 13.7 (U/mg) compared to 69 ± 3.2 (U/mg) for the commercial standard (Enzo Life Sciences, #SE-345). We also tested the preps of His-actS6K1 using the Kinase-Glo assay with a synthetic rpS6 peptide as the substrate (Fig. 3C). This assay monitors the activity of purified kinases by quantifying ATP consumption. We conducted the Kinase-Glo assay for preps in the presence and absence of the peptide substrate and found that catalytic ATP consumption was dose-dependent, substrate-dependent and essentially free of non-specific ATP degradation. The study did not include mass spectrometry analysis of affinity-purified His-actS6K1 to determine phosphorylation levels; however, kinase activity assays suggested a high stoichiometry of phosphorylation at T229.

### Evaluation of His-actS6K1 specificity through inhibitor profiling

**Fig. 4:**
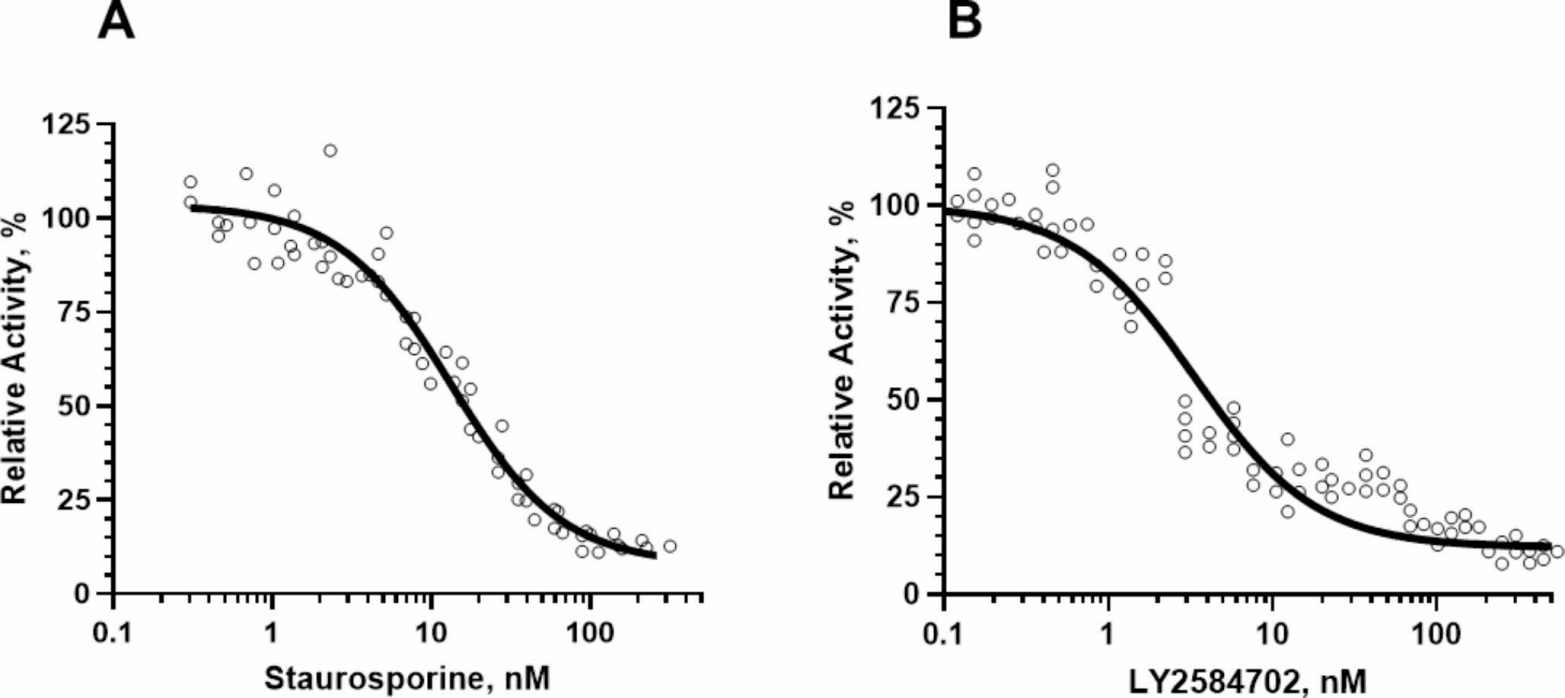
Inhibition of the His-actS6K1 catalytic activity by the pan-kinase inhibitor Staurosporine (**A**) and the S6K1-selective inhibitor LY2584702 (**B**) tested using the AlphaScreen assay. The results were obtained from four independent experiments conducted in triplicate. Dose-response curves and statistical analysis were performed using GraphPad Prism 9

To further verify the specificity of the recombinant protein, we analysed the sensitivity of purified His-actS6K1 to both nonspecific and specific S6K1 inhibitors: Staurosporin and LY2584702, respectively. As shown in Fig. 4, both compounds effectively inhibited His-actS6K1.

The biotinylated rpS6 peptide substrate was primarily designed for an AlphaScreen platform-based S6K1 assay, which was intended for high-throughput screening of S6K1 inhibitors. We used the purified His-actS6K1 (Prep #1) to optimize the parameters for both the reaction and the development steps of the AlphaScreen S6K1 assay. The assay demonstrated high sensitivity of His-actS6K1 to the tested inhibitors, including the pan-kinase inhibitor Staurosporine and the model S6K1-specific inhibitor LY2584702 (Merck) (Fig. 4).

The primary data from four independent experiments were processed using GraphPad Prism 9. The calculated IC50 values for Staurosporine and LY2584702 were 12.8 nM, (95%CI = 10.8 to 14.9) and 4.2 nM, (95%CI = 3.6 to 4.9) respectively. The Staurosporine IC50 measured by the AlphaScreen assay for His-actS6K1 was consistent with the reported ranges of 125.6 nM, 64.1 nM, and 9 nM from other studies [23] and commercial suppliers (Promega, #V4030; Eurofins, #2883). The wide range of Staurosporine IC50 values is understandable, given the differences in assay platforms, enzyme sources, substrates, and other variables used in the tests. Therefore, we were pleased to find that the IC50 for the inhibition of His-actS6K1 activity by the S6K1-selective inhibitor LY2584702 was very close to the value reported by the supplier (~ 4 nM, Merck, #SML2892).

## Discussion

The main aim of our research was to develop a versatile method for the expression and purification of highly active p70S6K1, suitable for high throughput enzymatic assays. Currently, several options exist for obtaining recombinant S6K1 in a highly active state. For example, commercially available active S6K1 (Thermo Fisher Scientific, #PV3815) was first expressed from the baculovirus system and then activated by in vitro phosphorylation at T229 by recombinant PDPK1. Highly active S6K1 has also been produced from transiently transfected mammalian cell cultures [20]. Despite the high quality of the enzymes obtained through such approaches, the methodology used is complicated, laborious, and expensive. To mention, phosphomimetic mutations at the T229 site of the activation loop (T229E) or combined mutations (T389E and T229E) do not replicate the activation achieved through enzymatic phosphorylation at T229 in S6K1, with T229E mutants exhibiting approximately 20% of the wild-type protein’s enzymatic activity when transiently expressed in COS-7 cells [24].

The baculovirus-mediated expression of highly active recombinant p70S6K1 obtained via co-infection with recombinant PDPK1 has been reported and well characterized by Keshwani et al. [21, 22]. Previously, we have tested the expression of active S6K1 via co-infection with the PDPK1 carrying virus (data not shown). We found that the quality and yield of S6K1 produced by co-infection were highly dependent on the optimal ratio of the viruses and the total MOI, both of which varied with different virus stocks. To address this variability, we produced a single construct that co-expressed both S6K1 and PDPK1 proteins. Co-expression of multiple genes from a single baculovirus was predominantly used for production of VLP (virus-like particles), heavy and light chains of antibodies, and multiprotein complexes [25, 26]. Co-expession of the protein kinases in Bac-to-Bac system was predominantly focused on regulatory subunits, chaperons or binding partners [27–29]. However, there are very few publications regarding the co-expression of protein kinases for activation purposes using the pFasBac™ Dual vector [30–32]. These publications primarily describe the use of PDPK1 co-expression for either regulatory studies or the production of activated kinases.

In our research we were the first to successfully demonstrate the expression of activated p70S6K1 using the pFastBac™ Dual vector of the Bac-to-Bac baculovirus expression system. The developed protocol facilitates the easy and efficient purification of recombinant S6K1, which is expressed in significant abundance, ensuring streamlined isolation for further research and applications. The effective targeting of recombinant His-actS6K1 by both selective and non-selective inhibitors suggests that the purified protein is well-suited for subsequent enzymatic studies, particularly for identifying potential S6K1 inhibitors through in vitro kinase assays.

## Conclusions

In summary, we found that the co-expression of *RPS6KB1* and *PDPK1* genes, driven by a single Bac-to-Bac baculovirus expression vector, provides a reliable and reproducible high-yield source of p70S6K1 with a high catalytic activity. Utilizing the developed baculovirus expression vector system and the associated expression/purification protocol, we isolated the activated kinase domain with an activity and purity sufficient for several enzymatic assay platforms. The conducted tests yielded consistent and reproducible results in immunoblotting, Kinase-Glo, and AlphaScreen assays. The range of in vitro kinase reactions provide an additional proof that purified recombinant His-actS6K1 is more active compared to the commercial, and therefore it is more efficient at phosphorylation its substrate. The AlphaScreen assay was optimized for a 384-well plate format, and was later used to test two model S6K1 inhibitors. Our analysis revealed that the assay is highly sensitive, leading us to conclude that the produced recombinant S6K1 is well-suited for the developed AlphaScreen assay and can be utilized in further high-throughput screening projects.

## Electronic supplementary material

Below is the link to the electronic supplementary material.

Supplementary Material 1

Supplementary Material 2

Supplementary Material 3

## Data Availability

No datasets were generated or analysed during the current study.
