# Supplementary material for: Co-expression of the RPS6KB1 and PDPK1 genes for production of activated p70S6K1 using bac-to-bac baculovirus expression system
Source: Mol Biol Rep. 2025 Jan 17;52(1):130. doi: 10.1007/s11033-024-10136-0 (PMC11742003; doi:10.1007/s11033-024-10136-0)
Supplement: Supplementary file 1 — Supplementary Material 1 [file 11033_2024_10136_MOESM1_ESM.docx]

| **AZ001** | GCCACCATGGACGGCACTGCAGCCGAG | Forw: PDPK1 | (aa51 isoform1); NcoI |
| --- | --- | --- | --- |
| **AZ002** | AGCAGCTGTCAGGTGAGCTTCGGAGGCGTC | Rev: PDPK1 | (aa359 isoform1); PvuII |
| **AZ003** | GGGGATCCGCCACCATGGCGCACCATCACCATCACCATGAAAACTTGTACTTTCAAGGCCCGGGAGTGTTTGACATAGACCTG | Forw: S6K1 | (aa3 isoformX), 6xHis, TEV; BamHI |
| **AZ005** | CTGAATTCAACTTTCAAGTACAGATGGAGCC | Rev: S6K1 | (aa398 isoformX); EcoRI; |

**Table S1. The Primers Used for Cloning.**
