# Supplementary material for: Co-expression of the RPS6KB1 and PDPK1 genes for production of activated p70S6K1 using bac-to-bac baculovirus expression system
Source: Mol Biol Rep. 2025 Jan 17;52(1):130. doi: 10.1007/s11033-024-10136-0 (PMC11742003; doi:10.1007/s11033-024-10136-0)
Supplement: Supplementary file 2 — Supplementary Material 2 [file 11033_2024_10136_MOESM2_ESM.docx]

**Table S2. Protein Purification Parameters.**

| **Prep #** | **Yield (mg/L)** | **Protein Stock Concentration (mg/ml)** | **Enzymatic Activity** | |
| --- | --- | --- | --- | --- |
|  |  |  | **U/mg** | **±SEM** |
| **1** | 6.1 | 0.48 | 166.2 | 9 |
| **2** | 5.4 | 0.38 | 129.8 | 24.2 |
| **3** | 4.0 | 0.35 | 117.7 | 8 |
| **Average** | 5.2 | - | 137.9 | 13.7 |
| **Commercial standard** | **-** | **0.1** | **69** | **3.2** |
