# Supplementary figures and images for: Co-expression of the RPS6KB1 and PDPK1 genes for production of activated p70S6K1 using bac-to-bac baculovirus expression system
Source: Mol Biol Rep. 2025 Jan 17;52(1):130. doi: 10.1007/s11033-024-10136-0 (PMC11742003; doi:10.1007/s11033-024-10136-0)

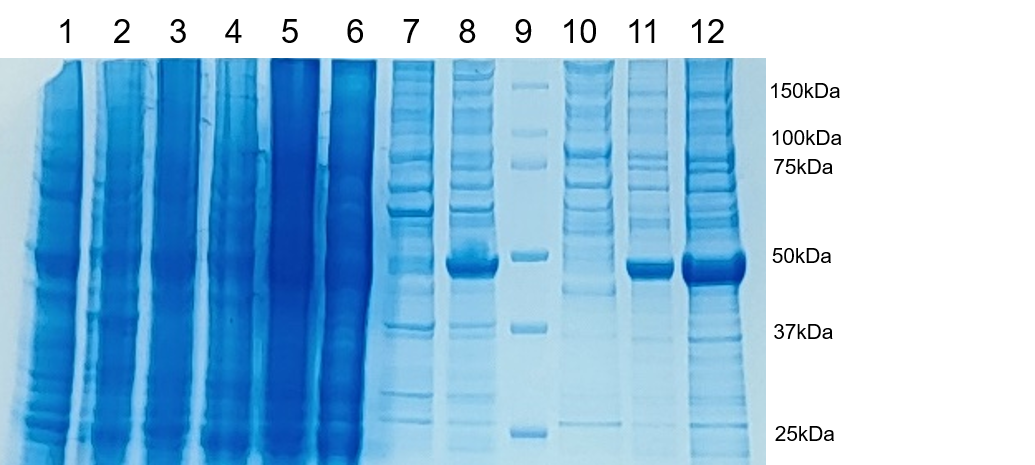

Supplement: Supplementary file 3 — Supplementary Material 3 [file 11033_2024_10136_MOESM3_ESM.tif]
